# Catastrophic antiphospholipid syndrome in lupus-associated immune thrombocytopenia treated with eltrombopag A case series and literature review

**DOI:** 10.1097/MD.0000000000032949

**Published:** 2023-02-10

**Authors:** Wakar Garra, Or Carmi, Shaye Kivity, Yair Levy

**Affiliations:** a Department of Internal Medicine E, Meir Medical Center, Kfar Saba, Israel; b Sackler School of Medicine, Tel Aviv University, Tel Aviv, Israel; c Rheumatology Unit, Meir Medical Center, Kfar Saba, Israel.

**Keywords:** catastrophic antiphospholipid syndrome, eltrombopag, immune thrombocytopenic purpura, lupus

## Abstract

Eltrombopag, a thrombopoietin receptor (TPO-R) agonist, is considered a second-line treatment for patients with refractory immune thrombocytopenia (ITP). Systemic lupus erythematosus (SLE) is frequently associated with ITP. In some cases, thrombocytopenia in SLE patients is attributed to concurrent antiphospholipid antibodies (APLA). Currently, data regarding treatment with TPO-R agonists for ITP in SLE or APLA patients are limited. The incidence of SLE flare or antiphospholipid syndrome while on TPO-R agonists has not been well-studied.

**Cases:**

We report 2 cases of female patients with SLE and concurrent triple positive APLA, without thrombotic events in their medical history, in our rheumatology clinic, who were treated for refractory ITP with eltrombopag. Both developed catastrophic antiphospholipid syndrome a few weeks after beginning treatment with eltrombopag. They were admitted to the intensive care unit and treated with solumedrol, plasmapheresis, anticoagulation and rituximab.

**Conclusions:**

We describe a severe possible side-effect of eltrombopag as a trigger of catastrophic antiphospholipid syndrome, a rare initial manifestation of antiphospholipid syndrome, in SLE patients with APLA. We suggest that APLA should be tested before initiating eltrombopag in patients with SLE-associated ITP. The safety of this treatment should be considered in these cases.

## 1. Introduction

Immune thrombocytopenia (ITP) is an acquired antibody-mediated immune disorder characterized by inadequate platelet production and increased apoptosis. It can be a primary condition or associated with other disorders, such as connective tissue diseases, including systemic lupus erythematosus (SLE), antiphospholipid syndrome (APS), and Felty syndrome.^[1,2]^

Thrombocytopenia has a prevalence of 7% to 30% among SLE patients. In some SLE patients, it is chronic and usually asymptomatic, whereas in others, it may be acute and severe, as a manifestation of disease exacerbation. Thrombocytopenia is also associated with antiphospholipid antibodies, which should be tested in all patients with SLE.^[1,2]^

The mechanism of ITP in SLE and APS is thought to be due to autoantibody-mediated peripheral platelet destruction and splenic sequestration, as well as decreased platelet production due to autoantibodies targeted against megakaryocytes.^[3]^

The decision to treat thrombocytopenia is based on several factors, including platelet count, bleeding risk, and active bleeding. A platelet count < 30,000 to 50,000/mm^3^ will usually require treatment, as bleeding risk increases. Glucocorticoids are the first-line therapy. Intravenous immunoglobulin (IVIG) can be added in cases of glucocorticoid-refractory thrombocytopenia or life-threatening bleeding. Danazol and hydroxychloroquine (Plaquenil) have been shown to improve the rate of sustained response. Immunosuppressive agents, such as azathioprine, mycophenolate, cyclophosphamide, cyclosporine or vincristine and splenectomy have been used as second-line treatments. The newer, biological agents that have been studied are rituximab, belimumab, and thrombopoietin (TPO) mimetics.^[1,4]^

In 2008, 2 novel TPO mimetics, eltrombopag (Revolade, Promacta) and romiplostim (NPlate) were approved for use for patients with chronic ITP who were refractory to conventional therapy.^[3,5]^

Eltrombopag is an oral nonpeptide TPO-R agonist, which binds to transmembrane sites on the TPO-R; thereby, allowing proliferation and differentiation of megakaryocytes and stimulated platelet production via the JAK/STAT signaling pathway.^[3,5]^

Thrombopoietin receptor agonist treatment for primary ITP has demonstrated clinical efficacy, with sustained improvement of platelet count in 70% to 80% of cases.^[6]^ While limited case reports and case series have shown promising outcomes for the efficacy or safety of these agents in patients with secondary ITP related to SLE and APS,^[3,7–10]^ other case reports have shown contradictory outcomes and reported thrombotic events in patients with antiphospholipid antibodies (APLA) treated with these agents.^[5,6,11–15]^ Information regarding the incidence of thrombotic events and autoimmune disease flares in SLE or APS patients treated with TPO-R agonists is limited.

We describe our experience with eltrombopag as a treatment for ITP in SLE patients with APLA and review the literature for catastrophic antiphospholipid syndrome (CAPS) as a potential severe side-effect of eltrombopag. We report 2 cases in our rheumatology clinic with SLE and concurrent triple positive APLA, with no thrombotic events in their medical history who were treated for secondary ITP with eltrombopag. Both developed CAPS a few weeks after beginning this treatment.

## 2. Cases

### 2.1. Case 1

A 24-year-old-female with a history of SLE, ITP, and triple positive APLA and mild aortic and mitral valve insufficiency. She had been diagnosed with SLE 8 years earlier, presenting with 5 of the 11 American College of Rheumatology 2019 classification criteria,^[16]^ including thrombocytopenia < 100 k, arthritis, lupus nephritis compatible with class II in renal biopsy, elevated anti-dsDNA antibody titers and APLA. She has been treated with Plaquenil. She had undergone several treatments for refractory ITP, including steroids, rituximab, and Imuran and IVIG. Two weeks before admission, 50 mg of eltrombopag daily had been initiated. Subsequently, 4 days prior to admission, eltrombopag was discontinued due to abdominal pain and vomiting related to its administration and gastrointestinal symptoms were relieved. The patient presented to the emergency department with general weakness and fatigue over the previous week. She had no fever, arthralgia, rash, oral ulcers, respiratory, and gastrointestinal or urinary complaints at presentation. Upon admission, she was tachycardic with a heart rate 140 beat/min, other vital signs were normal. Physical examination revealed diffuse pulmonary crackles on auscultation, other physical findings were normal. An electrocardiogram (ECG) demonstrated sinus tachycardia, 150 beats/minute with ST segment depressions in V2-6. No ST segment elevations were found in posterior ECG. Her laboratory evaluation showed elevated white blood cell (WBC) count 13 × 10^9^ /L, hemoglobin 13.6 g/dL, normal platelet count, elevated AST 182 U/L and ALT 95 U/L and acute prerenal kidney injury with creatinine 1.9 mg/dL (baseline 0.9 mg/dL) and urea 92 mg/dL. C-reactive protein was 13 mg/dL. Due to the changes detected on ECG, troponin level was measured at > 10,000 ng/L. D-Dimer was 1507 ng/mL, fibrinogen 648 mg/dL, prothrombin time 12.6 seconds and partial thromboplastin time was normal. Blood smear revealed no schistocytes. Chest X-ray demonstrated bilateral pulmonary infiltrates. Echocardiography revealed moderate left ventricle (LV) dysfunction and moderate mitral regurgitation with no pericardial effusion.

As multi-organ involvement was present in a triple positive APLA patient, CAPS was suspected. The patient was immediately given a pulse of 1 gram solumedrol. CT angiography of the chest demonstrated consolidation on the left lower lobe and ground glass opacity infiltrations and consolidations in both lungs with no pulmonary embolism. Differential diagnosis of the radiographic findings included atypical pneumonia in an immunodeficient patient due to prolonged steroid treatment, COVID-19 infiltrates, pulmonary congestion, acute respiratory distress syndrome and diffuse alveolar hemorrhage. PCR test for COVID-19 was negative. Several hours later, in the emergency department, altered mental status was noticed. The patient was tachycardic and dyspneic with O_2_ saturation 90% on room air and 99% on nasal cannula, temperature was 38.6°C and blood pressure was 138/68 mm Hg. A noncontrast head CT was normal. Lumbar puncture was performed, which excluded central nervous system infection. The patient was hospitalized in the intensive care unit (ICU).

Several hours later, dyspnea increased with no improvement on high-flow nasal cannula and emergent intubation ensued. Peri-intubation cardiac arrest occurred with successful resuscitation. Based on the diagnosis of probable CAPS, in a multi-disciplinary decision, triple treatment with steroids, anticoagulation and plasmapheresis was immediately initiated. Sepsis, pneumonia and SLE flare were suspected as triggers for CAPS. Blood cultures were drawn and antibiotic therapy with rocephin, resprim and azenil was started. The anti-ds DNA antibody titers and complement levels were normal. Transesophageal echocardiography revealed preserved LV wall motion, regional LV wall motion abnormality with inferobasal and posterobasal hypokinesis, Libman- Sacks endocarditis, moderate mitral regurgitation, severe aortic regurgitation and small pericardial effusion. The patient was not a candidate for coronary angiography due to high-risk of thrombosis as a consequence of active CAPS. She was treated with intravenous furosemide. On the third day of hospitalization, laboratory evaluation revealed a 4 g/dL decrease in hemoglobin relative to its initial value at presentation and worsening creatinine at 2.3 mg/dL. Repeat chest X-ray showed increased pulmonary infiltrates. Diffuse alveolar hemorrhage was more likely. Renal Doppler ultrasound was normal. CT angiography of the brain demonstrated bilateral hypodense lesions in the frontal and occipital lobes, cerebellum and brain stem, compatible with subacute infarcts. The patient was treated with Solumedrol 500 mg daily, 2 courses of 1 gram rituximab and 10 courses of plasmapheresis. It was difficult to achieve a therapeutic PTT while on heparin. The patient developed left brachial artery thrombosis. Anticoagulation was converted to Angiomax with rapid clinical and laboratory improvement. She was successfully extubated. Blood cultures showed no evidence of infection. We suggest that CAPS was triggered in our patient as a result of eltrombopag administration. The patient was discharged home after a month of hospitalization with the recommendation to continue Plaquenil, prednisone with tapering down and subcutaneous clexane injections.

### 2.2. Case 2

A 52-year-old-female with a history of hypertension, diabetes mellitus, moderate mitral regurgitation, SLE, ITP, and triple positive APLA. She had been diagnosed with SLE several years earlier, presenting with 5 of the 11 American College of Rheumatology 1997 classification criteria,^[17]^ including thrombocytopenia < 100 K, arthritis, high antinuclear antibody count, elevated anti-dsDNA antibody titers, and APLA. She was treated for many years with Plaquenil that was eventually discontinued due to retinal toxicity. She had undergone several treatments for refractory ITP, including steroids, rituximab, and IVIG. Three weeks before admission, the patient had begun to receive 50 mg of eltrombopag daily. She presented to the emergency department with general weakness, shortness of breath and epigastric abdominal pain that started 2 days before. The patient denied fever, cough, chest pain, vomiting or diarrhea. She did not note any changes in urinary frequency or color. Vital signs on admission were blood pressure 190/85 mm Hg, heart rate 60/minute, respiratory rate 28/minute, O_2_ saturation 92% on room air, 95% with nasal cannula. Her physical examination revealed, bibasilar pulmonary crackles, systolic heart murmur on the aortic point, no tenderness on abdominal palpation, lower extremity edema and diffuse livedo reticularis rash. Hemoglobin was 12 g/dL, WBC count 6 × 10^9^ /L and platelet count 18 × 10^9^ /L.

Laboratory evaluation also showed acute renal failure with creatinine 6.2 mg/dL and urea 150. C-reactive protein was 200 mg/L, troponin was 1.7 mcg/L. Partial thromboplastin time was prolonged but prothrombin time and fibrinogen were normal. ECG demonstrated sinus rhythm, 60/minute, without ischemic changes. Chest X-ray demonstrated bilateral pulmonary infiltrates. The differential diagnosis included flash pulmonary edema due to malignant hypertension with organ damage to kidney and heart; pulmonary edema due to lupus flare with heart, lung and kidney involvement; pulmonary edema due to heart failure, valvulopathy or as part of volume overload due to renal failure; bilateral atypical pneumonia; and acute respiratory distress syndrome secondary to sepsis. With both thrombocytopenia and acute renal failure, atypical hemolytic uremic syndrome was also considered. No schistocytes were detected in blood smear. Treatment was started with intravenous Solumedrol as well as intravenous broad-spectrum antibiotics, furosemide and antihypertensive drugs. No improvement was seen. The patient persisted with dyspnea and malignant hypertension. Echocardiography demonstrated preserved right and LV wall motion with an estimated ejection fraction of 60%, moderate aortic stenosis (AS), severe aortic regurgitation (AR), minimal AR and tricuspid regurgitation (TR), no pulmonary hypertension and small pericardial effusion. With a diagnosis of probable catastrophic APS (heart, kidney, and lung involvement), the patient was transferred to the ICU, where she was intubated. Treatment with Solumedrol was continued along with plasmapheresis and anticoagulation with angiomax. Anti ds-DNA antibody titer and complement level were normal. It should be noted that the patient previously had elevated anti ds-DNA antibody titer. Urine sedimentation revealed no dysmorphic RBC casts but granular casts, so glomerulonephritis was less likely. The patient was not a candidate for kidney biopsy. Doppler ultrasound and magnetic resonance angiogram of renal arteries were normal. No pulmonary embolism was found in lung ventilation-perfusion scan. No evidence of infection was found in blood cultures. With the triple treatment of steroids, plasmapheresis and anticoagulation, the patient improved dramatically and was extubated. Later, during her stay in the ICU, thrombocytopenia worsened while on steroids and plasmapheresis. Therefore, in a multi-disciplinary decision, IVIG treatment along with platelet transfusions were initiated. Nevertheless, no improvement in the thrombocytopenia was observed and splenectomy remained the last resort to treat thrombocytopenia. The patient underwent rehabilitation and thereafter was discharged home with prednisone and subcutaneous Clexane injections. Coumadin was advised due to the instability of the platelet count and the risk for bleeding.

## 3. Discussion

ITP is an acquired autoimmune disease characterized by the destruction and inadequate production of platelets that is mediated by antibodies. It may be primary or secondary to an underlying disorder – infections, autoimmune diseases, cancer or medications.^[4,11]^

Thrombocytopenia occurs in 7% to 30% of patients with SLE. Up to 25% have thrombocytopenia < 100,000/mm^3^. Thrombocytopenia is also associated with APLA, which should be tested in all patients with SLE. It occurs in 22% to 42% of APS patients.^[1,2,18]^

Standard first-line ITP treatment continues to be steroids. IVIGs are usually reserved to treat hemorrhagic cases. Other second line treatments include azathioprine, mycophenolate, cyclophosphamide, cyclosporine, and rituximab. In refractory or relapsing situations, splenectomy is the traditional second-line option as it is the only curative treatment for ITP. Hydroxychloroquine has been shown to improve the rate of sustained response.^[4]^

Conversely, secondary ITP is often refractory to steroids and splenectomy, reflecting that it is the outcome of underlying disease.^[5,7]^

Romiplostim and eltrombopag are both TPO-R agonists that were approved in 2008 by the United States Food and Drug Administration and the European Medicines Agency to treat patients with insufficient response to conventional therapy. Results of clinical trials showed 80% efficacy in achieving hemostatic platelet levels. Data regarding its efficacy and safety for secondary ITP, such as SLE and APS-related ITP are sparse.^[3,7–9,11,12]^

Eltrombopag is an oral, small molecule, nonpeptide TPO-R agonist. It promotes the proliferation and differentiation of megakaryocytes in bone marrow, resulting in a dose-dependent increase in normally functioning platelets.^[7,13]^

Toxicities related to eltrombopag use, reported in the literature include bone marrow fibrosis, rebound thrombocytopenia, hematologic malignancies and thrombosis.^[13]^ It is approved to treat thrombocytopenia secondary to aplastic anemia, hepatitis C, chronic lymphocytic leukemia, SLE, Evans syndrome, human immunodeficiency virus, and celiac disease among patients who experienced loss of response to standard treatment for ITP.^[5]^

Early case reports and case series describing the use of TPO mimetics in SLE patients, including those with APS, largely showed that these agents were effective and safe without significant risks, and especially for thrombosis.^[3,7–10]^ More recently, several reports have noted potential thrombotic risks of these agents in SLE patients, particularly those with APLA.^[5,6,11–15]^

In this article, we report 2 cases of patients with SLE and triple positive APLA, complicated by CAPS related to eltrombopag administration, a few weeks after treatment onset. The first patient presented with normal platelet count, while the second presented with severe thrombocytopenia. CAPS as an initial manifestation of APS is very rare, occurring in 1% of APS cases. Mortality related to CAPS may reach 50%, despite treatment.

After a systematic review of the literature, we summarize the following studies and case reports, which indicated that TPO-R agonists are associated with thrombotic risk in SLE patients or in patients with APLA.

Tomov et al^[12]^ (2013) reported a case of a 19-year-old-female with a history of SLE and negative serology for APLA, who developed acute renal failure 6 weeks after initiation of Romiplostim. Kidney biopsy revealed thrombotic microangiopathy. The patient presented with thrombocytopenia 60 K/μL.

LaMoreaux et al^[13]^ (2016) described 1 pediatric and 1 adult patient, with antiphospholipid antibodies, 1 with SLE who developed CAPS 1 month after treatment with the TPO-R agonist Romiplostim. The first patient presented with normal platelet count while the second had thrombocytopenia 111,000 K/μL.

Borrell et al^[15]^ (2016) reported a case of an adult patient with SLE who developed deep venous thrombosis 3 months after Romiplostim was initiated. Platelet count was 177,000/mm^3^. Anticardiolipin and beta 2 glycoprotein antibodies were positive.

Boulon et al^[14]^ (2016) described a 61-year-old-female with a history of APS, with positive lupus anticoagulant. Anti-GPIIb IIIa antibodies were positive. She developed pulmonary embolism 1 month later, despite low platelet count 119,000/mm^3^ and an international normalized ratio 4.97 above the therapeutic value.

Gonzalez-Lopez et al^[5]^ (2017), in a multi-center retrospective cohort study that included 87 secondary ITP patients (46 secondary to autoimmune syndromes) who had been treated with eltrombopag were evaluated. Secondary ITP of autoimmune syndromes included SLE (13), Evans syndrome (8), Sjogren syndrome (4), rheumatoid arthritis (3), autoimmune hepatitis (2), primary biliary cirrhosis (2), psoriatic arthritis (1), Graves’ disease (1), inflammatory bowel disease (1). Four patients with ITP secondary to immune disorders had thrombotic complications, including superficial phlebitis (1), deep venous thrombosis (1), pulmonary embolism (1) and ischemic stroke (1). None had thrombocytosis at the time of their thrombosis episode. Two patients received eltrombopag 50 mg/day, 1 patient 75 mg/day and 1 patient 100 mg per day.

Guitton et al^[6]^ (2018), in a multi-center retrospective cohort study including 18 patients with SLE-ITP treated with TPO-R agonists, 55% (10 patients) had APLA and 27% (5 patients) had definite APS. Six patients (33%) received Romiplostim, 5 (28%) eltrombopag and 7 (39%) both, sequentially. After a median follow-up of 14.7 months with TPO-R, 5 patients experienced thrombotic events (TE) during treatment with eltrombopag (n = 4) or Romiplostim (n = 1), at a median of 3.5 months after treatment onset (range 1–7 months). Platelet count at the time of the TE was > 150 × 10^9^ /L (range 166–484 × 10^9^ /L). Four arterial TEs occurred in 4 patients who received eltrombopag, with previous APS in 3 and with APLA positivity without definite APS in the last patient. The arterial TEs were 2 myocardial infarctions, 1 stroke and CAPS that occurred 6 months after initiation of TPO-R agonists. Two venous TEs, pulmonary embolism and a year later, intracranial sinus thrombosis, occurred in a patient without APS or APLA positivity who received Romiplostim. No SLE flare was observed in patients exposed to TPO-R antagonists.

Gonzalez-Lopez et al^[11]^ (2019) conducted a retrospective cohort study including adult patients ≥ 65-years-old, treated with eltrombopag, with 106 primary ITP patients and 39 secondary ITP patients (20 secondary to immune disorders, 7 to infectious diseases and 12 to lympho-proliferative disease). One transient ischemic attack in a newly diagnosed ITP patient and 2 pulmonary embolisms in secondary ITP patients (1 with APS and 1 with chronic lymphocytic leukemia), 12 and 8 months after starting eltrombopag with platelet counts of 263 and 158 × 10^9^ /L, respectively.

As noted from our case series and the case reports and studies mentioned above, we deduce that thrombosis in lupus and APLA patients may occur regardless of the time since eltrombopag initiation, platelet count during the disease course or eltrombopag dose. In some cases, it may be further complicated with CAPS, a highly fatal complication.

Patients with SLE are at increased risk for thrombosis, both arterial and venous, regardless of their APLA status.^[1,2]^ Some epidemiologic studies have suggested that patients with ITP are at increased risk for developing arterial and venous thrombosis despite thrombocytopenia,^[19]^ as a result of accelerated atherosclerosis. Therefore, it is not surprising that administering TPO-R agonists to patients with preexisting thrombotic risk factors may lead to significant thrombotic morbidity.

In mouse models, TPO-R agonists have shown increased peripheral white blood cell counts. This may contribute to autoimmune and result in autoimmune disease flares.^[20]^

Our case series demonstrates that eltrombopag use in SLE or APLA patients may be associated with a potentially devastating thrombotic adverse event, CAPS, independently of platelet count, time from initial administration, and dose. A decision to start eltrombopag should be part of a multi-disciplinary plan. Prior to treatment, patients must be tested for antiphospholipid antibodies.

## Author contributions

**Investigation:** Wakar Garra, Or Carmi, Shaye Kivity, Yair Levy.

**Resources:** Wakar Garra, Or Carmi, Shaye Kivity, Yair Levy.

**Supervision:** Wakar Garra, Yair Levy.

**Visualization:** Wakar Garra, Yair Levy.

**Writing – original draft:** Wakar Garra, Yair Levy.

**Writing – review & editing:** Wakar Garra, Yair Levy.
